# Differences in gray matter structure correlated to nationalism and patriotism

**DOI:** 10.1038/srep29912

**Published:** 2016-07-15

**Authors:** Hikaru Takeuchi, Yasuyuki Taki, Atsushi Sekiguchi, Rui Nouchi, Yuka Kotozaki, Seishu Nakagawa, Carlos Makoto Miyauchi, Kunio Iizuka, Ryoichi Yokoyama, Takamitsu Shinada, Yuki Yamamoto, Sugiko Hanawa, Tsuyoshi Araki, Hiroshi Hashizume, Keiko Kunitoki, Yuko Sassa, Ryuta Kawashima

**Affiliations:** 1 Division of Developmental Cognitive Neuroscience, Institute of Development, Aging and Cancer, Tohoku University, Sendai, Japan; 2 Division of Medical Neuroimaging Analysis, Department of Community Medical Supports, Tohoku Medical Megabank Organization, Tohoku University, Sendai, Japan; 3 Department of Radiology and Nuclear Medicine, Institute of Development, Aging and Cancer, Tohoku University, Sendai, Japan; 4 Department of Functional Brain Imaging, Institute of Development, Aging and Cancer, Tohoku University, Sendai, Japan; 5 Human and Social Response Research Division, International Research Institute of Disaster Science, Tohoku University, Sendai, Japan; 6 Smart Ageing International Research Center, Institute of Development, Aging and Cancer, Tohoku University, Sendai, Japan; 7 Graduate Schools for Law and Politics, The University of Tokyo, Bunkyo, Tokyo, Japan; 8 Japan Society for the Promotion of Science, Tokyo, Japan; 9 Faculty of Medicine, Tohoku University, Sendai, Japan

## Abstract

Nationalism and patriotism both entail positive evaluations of one’s nation. However, the former inherently involves derogation of other nations, whereas the latter is independent of comparisons with other nations. We used voxel-based morphometry and psychological measures and determined nationalism and patriotism’s association with gray matter density (rGMD) and their cognitive nature in healthy individuals (433 men and 344 women; age, 20.7 ± 1.9 years) using whole-brain multiple regression analyses and post hoc analyses. We found higher nationalism associated with greater rGMD in (a) areas of the posterior cingulate cortex and greater rGMD in (b) the orbitofrontal cortex, and smaller rGMD in (c) the right amygdala area. Furthermore, we found higher patriotism associated with smaller rGMD in the (d) rostrolateral prefrontal cortex. Post hoc analyses revealed the mean rGMD of the cluster (a) associated with compassion, that of (b) associated with feeling of superiority, that of (c) associated with suicide ideation, and that of (d) associated with quality of life. These results indicate that individual nationalism may be mediated by neurocognitive mechanisms in social-related areas and limbic neural mechanisms, whereas patriotism may be mediated by neurocognitive mechanisms in areas related to well-being.

A recent study showed that political orientation (conservativeness–liberalism) is underlain by human brain structures such as the anterior cingulate cortex (ACC) and amygdala^1^. However, human political opinions have axes other than conservativeness–liberalism. In particular, nationalism as defined below is an important factor that has pushed much of the world into chaos and war^2^. Most studies related to nationalism focus on two factors: nationalism and patriotism^3^,^4^,^5^, both of which are associated with conservativeness in the US^4^, although there are other parallel political opinions. It has been argued that the distinction between nationalism and patriotism is important^3^,^4^. There are several ways of defining nationalism and patriotism, but one states that nationalism is an identification with and a positive evaluation of one’s nation, which is inherently related to derogation of other nations^5^. In contrast, patriotism is defined as pride in one’s nation, which is based on a positive evaluation of the nation independently of comparisons with other countries^5^.

Nationalism and patriotism have distinct unique psychological characteristics, as follows. Nationalism is negatively associated with acceptance of the Euro^6^ in Austria and European identity^6^, and it is positively associated with xenophobia^7^; greater tolerance toward immigration in Switzerland^8^; support toward nuclear armament policies and readiness to go to war, but less willingness to risk ones’ life, as compared with patriotism^4^,^9^; militarism^10^; aggressiveness and competitiveness (weakly)^4^,^9^; and a wide range of psychological characteristics related to a lack of compassion, such as human right abuses of minorities and ethnic violence^11^. On the other hand, patriotism is positively associated with European identity^6^; greater tolerance in Switzerland^8^; a stronger early attachment to one’s father and feelings of belonging, security, and self-enhancement^2^,^4^,^9^; and a more cooperative or peaceful approach to the world^2^.

Thus, nationalism and patriotism underlie individual support for important policies, and nationalism is an important factor that pushes much of the world into chaos and war^2^. Nationalism and patriotism are likely to have an innate biological nature because with political conservatism, which has an essential association with nationalism and patriotism^12^, genetics accounted for approximately half of the variance^13^. And political conservatism has also already been shown to be associated with regional gray matter structures^1^. For more discussion regarding legitimacy of investigating correlations of nationalism and patriotism with anatomical structure, see Supplemental Discussion.

Therefore, it is important to determine the brain characteristics that underlie individual nationalism and patriotism so that they can be better understood. However, despite the unique importance of nationalism and patriotism, their anatomical basis is unknown.

In general, as described above, lower nationalism appears to be associated with a wide range of psychological characteristics related to (a) aggressiveness and competitiveness, (b) lack of compassion and cooperativeness, and (c) feelings of superiority over others; whereas patriotism is associated with a wide range of psychological characteristics related to (d) positive feeling and (b) cooperativeness. Previous neuroimaging studies associated several areas with aggression, but among them, the amygdala is believed to play a central role in social aggression and negative emotional characteristics, possibly via an excessive response in this area (for review, see ref. 14). However, we previously showed that brain structures in the dorsal ACC and the fronto-polar cortex were associated with positive feelings (quality of life; (QOL)^15^. Perhaps related to this, fronto-polar cortex is associated with pro-social (tendency to help others and be positive toward others) sentiments^16^. As described above, structures in ACC and the amygdala have been associated with individual liberalism and conservativeness^1^. Another study showed moral behaviors that have roots in-group loyalty (which may be associated with nationalism or patriotism), is associated with insula structure^17^. Pro-social cognitive traits such as empathy related cognitive traits have been associated with brain structures in the medial prefrontal cortex and posterior cingulate cortex (PCC)^18^,^19^,^20^. Functional neuroimaging studies demonstrated that the orbitofrontal cortex (OFC) is particularly important for social comparisons^21^. Finally, from another perspective, functional imaging studies of in group cognition suggest, these areas are generally associated with patriotism and nationalism. These studies (for review, see ref. 22) have suggested medial prefrontal cortex is associated with categorization of in-group members, regions such as the ACC, insula, medial prefrontal cortex are associated with empathy toward members within the groups and outside the groups. And face perception of the outgroup member is associated with the amygdala and such activation is associated with implicit attitude toward outside members. Therefore, we hypothesized that individual nationalism and patriotism would be associated with differences in the structures in these areas. However, given the complexity of the nature of cognitive functions involved in these traits, other areas may not be able to be excluded from the candidates, which led to the requirement for an exploratory whole-brain search. On the other hand, our strong a priori focus was set on the amygdala and ACC, where regional gray matter (GM) structure was associated with individual liberalism and conservativeness, which are essentially associated with patriotism and nationalism^12^.

To test our hypothesis, we investigated how individual differences in nationalism and patriotism were associated with the regional GM density (rGMD) using voxel-based morphometry (VBM)^23^. To assess nationalism and patriotism, we used the National Identity Scale^24^.

Using neuroimaging techniques, we determined whether these complex cognitions in our society are correlated with neural mechanisms in limbic areas, such as the amygdala, and as well as higher order neural mechanisms in areas, such as the rostrolateral prefrontal cortex (RLPFC). This is because the amygdala is related to several instinctive behaviors^25^ and underlies a wide range of biases such as racial bias^26^. Further, the rostrolateral prefrontal cortex (RLPFC), is related to well-being^15^.

## Methods

### Subjects

Seven hundred and seventy-seven healthy, right-handed individuals (433 men and 344 women; 20.7 ± 1.9 years) participated in this study as part of an ongoing project investigating associations among brain imaging, cognitive functions, aging, genetics, and daily habits. Data derived from the subjects in this study are to be used in other studies irrelevant to the theme of this study. Some of the subjects who participated in this study also became subjects of intervention studies (psychological and imaging data recorded before the intervention were used in this study). Psychological tests and MRI scans not described in this study were performed together with those described in this study. All subjects were university, college, or postgraduate students or subjects who had graduated from these institutions within 1 year before the experiment and had normal vision. None had a history of neurological or psychiatric illness. Handedness was evaluated using the Edinburgh Handedness Inventory^27^. Written informed consent was obtained from each subject in accordance with the Declaration of Helsinki (1991). This study was approved by the Ethics Committee of Tohoku University. All experiments were performed in accordance with the institutional guidelines. For the limitations of this study, including those of the participants’ characteristics, see Supplemental Discussion.

### National Identity Scale

The National Identity Scale^24^ was used to assess individual nationalism and patriotism. This questionnaire is a self-reported measure of Japanese national identity and has been used for measuring Japanese national identity. This scale employs a five-point Likert scale with a response format that ranges from “I agree” to “I disagree”. This scale probes various tendencies related to national identity using multiple factors. In the present study, we used two factors: nationalism and patriotism. The nationalism factors comprised six items (e.g., “The Japanese people are among the finest in the world,” and “Given Japanese economic superiority, it is only correct that we should have a bigger say in the United Nations and other international organizations”). The patriotism factors comprised seven items (e.g., “I love my country, Japan” and “I do not feel much attached to Japan” (reverse item). The item contents were quite similar to the items used to test patriotism and nationalism in questionnaires developed in foreign countries^4^,^9^ and agreed with the definition of nationalism and patriotism given in the Introduction. The answers to the questions related to each factor were compiled into a single score for each factor (the responses to the reverse items were added after calculating as 6−x). Higher scores indicated higher nationalism and patriotism. For information regarding the reliability and validity of this scale, see Supplemental Methods.

We state that there are no relevant political-associated measures that were not disclosed in this study, other than the National Identity Scale. For the rationale of this choice, see Supplemental Methods.

### Assessment of psychometric measures of general intelligence

Raven’s Advanced Progressive Matrix (RAPM)^28^ was used to assess intelligence^28^ and adjust for the effect of general intelligence on brain structures. For more details of how RAPM was performed, please refer to our previous studies^29^.

### Psychological measures assessed using questionnaires

We employed several questionnaires to assess the individual characteristics that we hypothesized would be associated with nationalism and patriotism. To assess cooperativeness and associated cognitive characteristics, a Japanese version^30^ of the Temperament Character Inventory^31^ was used, where we employed the cooperativeness scale and cooperativeness subscales: social acceptance vs social intolerance, empathy vs social disinterest, helpfulness vs unhelpfulness, compassion vs revengefulness, and pure-hearted principles (integrated conscience) vs self-serving advantage. To assess positive feelings, we used the average QOL measure of the Japanese version of the QOL scale, WHOQOL-26^32^. To assess negative feelings, we used the depression and suicide ideation subscales of the General Health Questionnaire 30^33^ and the score on the Beck Depression Inventory^34^. To assess feelings of superiority over others, we used the sense of superiority and competence obtained using the Narcissistic Personality Inventory^35^. To assess aggressiveness indirectly, we used the measure of trait anger. Trait anger can be easily assessed and fundamentally associated with aggressiveness and have common neural and molecular mechanisms^36^,^37^. For this purpose, we used trait anger subscale of the State-Trait Anger Expression Inventory^38^. For an explanation why we did not include these measures in the whole brain multiple regression analysis, see Supplemental Discussion.

### Image acquisition

All MRI data acquisition was performed using a 3-T Philips Achieva scanner. High-resolution T1-weighted structural images (T1WIs: 240 × 240 matrix, TR = 6.5 ms, TE = 3 ms, FOV = 24 cm, slices = 162, in-plane resolution = 1 × 1 mm, thickness = 1.0 mm) were collected using a magnetization-prepared rapid gradient echo sequence. For the introduction of other images obtained in this project, see Supplemental Methods.

### Preprocessing of T1-weighted structural data

Preprocessing of the structural data was performed using Statistical Parametric Mapping software (SPM8; Wellcome Department of Cognitive Neurology, London, UK) implemented in Matlab (Mathworks Inc., Natick, MA, USA). Using a previously described method^39^ involving a new segmentation algorithm implemented in SPM8, the diffeomorphic anatomical registration through exponentiated lie algebra (DARTEL) registration process implemented in SPM8, images were spatially normalized to the Montreal Neurological Institute (MNI) space to give images with 1.5 × 1.5 × 1.5 mm^3^ voxels. Subsequently, all images were smoothed by convolving them with an isotropic Gaussian kernel of 12 mm full width at half maximum (FWHM) for the reasons described below. For more details of these methods, and a general description of VBM, see Supplemental Methods.

### Statistical analyses of psychological data

Behavioral data were analyzed using SPSS 22.0 (SPSS Inc., Chicago, IL). The multiple regression analyses included age, sex, the RAPM score, and the nationalism and patriotism scores as covariates to test the association of nationalism and patriotism with 11 other psychological variables described in the *Psychological measures assessed using questionnaires subsection* of the Methods. In psychological analyses, results with a threshold of p < 0.05, corrected for false discovery rate (FDR) using the two-stage sharpened method^40^, were considered statistically significant. The correction for multiple comparisons using this method was applied to the results of the abovementioned 22 associations (nationalism, patriotism ×11 psychological variables).

### Statistical analyses of imaging data

We investigated rGMD associated with individual differences in nationalism and patriotism. Statistical analyses of morphological data were performed using VBM5 software, an extension of SPM5 for the reasons as described in Supplemental Methods. In the analyses, we included voxels that are likely to be gray matter to some extent. For details, see Supplemental Methods.

We investigated the association between rGMD and individual differences in nationalism and patriotism using the whole-brain multiple regression analysis. In this analysis, we performed a single whole-brain multiple regression analysis using sex, age, the RAPM score, the total intracranial volume (TIV; total GM volume + total WM volume + total cerebrospinal fluid volume), and the nationalism and patriotism scores on the National Identity Scale.

The statistical significance level was set at *P* < 0.05, corrected at the non-stationary cluster level^41^ with an underlying voxel level of *P* < 0.001. For rationale and introduction of this method, see Supplemental Methods.

Furthermore, for areas with a strong a priori hypothesis that are described in Introduction, namely the bilateral amygdala, the dorsal part of the ACC area, the statistical significance level was set at *P* < 0.05, with small volume correction for multiple comparisons (family-wise error) in regions of interests (ROIs). For details of constructions of these ROIs, see Supplemental Methods.

The post-hoc analyses using the mean rGMD value within the significant clusters identified through the abovementioned analyses and associated psychological variables were performed and results with a threshold of *P* < 0.05, uncorrected were considered statistically significant in these analyses.

In these analyses, the dependent variables were the mean rGMD values for these clusters and the independent variables were age, sex, the RAPM score, TIV, and one of the psychological variables that were hypothesized to be related to nationalism and patriotism, as described above. We did not include all personality variables as covariates in the multiple regression analyses because of the same reasons these were not included in the analyses of patriotism and nationalism. For details, see Supplemental Methods.

## Results

### Basic data

Table 1 shows the average and standard deviation (SD) results for age, RAPM scores, nationalism, and patriotism in males and females. Table 2 shows the distributions of the nationalism and patriotism scores in males and females.

A multiple regression analysis with the nationalism score as the dependent variable and age, sex, and the RAPM score as independent variables showed that males had significantly higher nationalism scores, which is consistent with a previous study^42^. However, the patriotism score was not associated with sex. After correcting for the effects of age and sex, the patriotism and nationalism scores were significantly positively correlated [*P* = 2.91*10^−27^, *t* = 11.24, standardized partial regression coefficient (β) = 0.377].

We also performed multiple regression analyses with age, sex, the RAPM score, and the nationalism and patriotism scores as independent variables, and any one of the following as dependent variables: average QOL score^32^, feelings of superiority score^35^, trait anger score^38^, cooperativeness score^30^, and cooperativeness subscores, i.e., social acceptance vs social intolerance, empathy vs social disinterest, helpfulness vs unhelpfulness, compassion vs revengefulness, and pure-hearted principles vs self-serving advantage (see Methods for details). With this model, we can see which of patriotism and nationalism was correlated (after correcting for the effect of the other) with the other psychological variables. The results showed that the nationalism score was significantly and positively correlated with the feelings of superiority score and trait anger score and was significantly and negatively correlated with the cooperativeness score and cooperativeness subscores. However, the negative association between the nationalism score and compassion vs revengefulness was very significant. The patriotism score was significantly and positively correlated with the average QOL and was significantly and weakly positively correlated with the social acceptance vs social intolerance score. However, neither nationalism nor patriotism was significantly correlated with negative emotions (Beck Depression Inventory score and suicide ideation and depressive tendency). The results of the statistical analyses are shown in Table 3.

### Correlations between rGMD, nationalism, and patriotism

**Figure 1:**
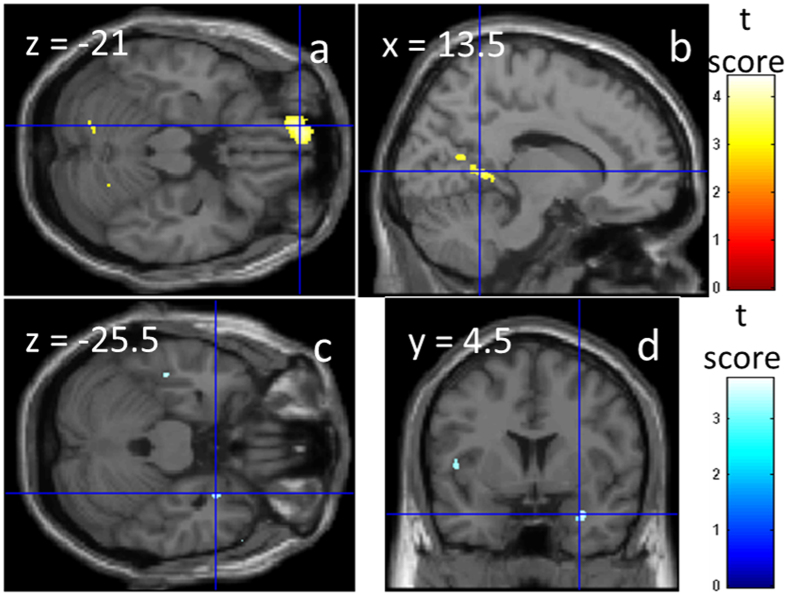
Anatomical correlates of nationalism. The results are overlaid on SPM5’s “single subject” T1-weighted structural image. Blue represents the T score of the negative correlation, and red represents the T score of the positive correlations. Results are shown with *P* < 0.001, uncorrected. (**a,b**) Positive and (**c,d**) negative rGMD correlates with nationalism. (**a**) A significant positive correlation is observed in the area of OFC. (**b**) A significant positive correlation is observed in the area of PCC. (**c,d**) A significant negative correlation is observed in the area of the right amygdala.

We investigated the association between rGMD and individual differences in nationalism and patriotism. For the rGMD correlates of nationalism, the whole-brain multiple regression analysis showed that nationalism was significantly positively correlated with rGMD of the anatomical cluster in OFC (Fig. 1a) and the anatomical cluster that mainly spread in PCC (Fig. 1b). The peak as well as most of the voxels of the cerebrum in the former cluster are included in PCC as defined by the Talairach Daemon option^43^ of the WFU PickAtlas Tool (http://www.fmri.wfubmc.edu/cms/software#PickAtlas). Small volume correction (SVC) revealed a significant negative correlation between the nationalism scores and rGMD in the right amygdala (Fig. 1c,d).

**Figure 2:**
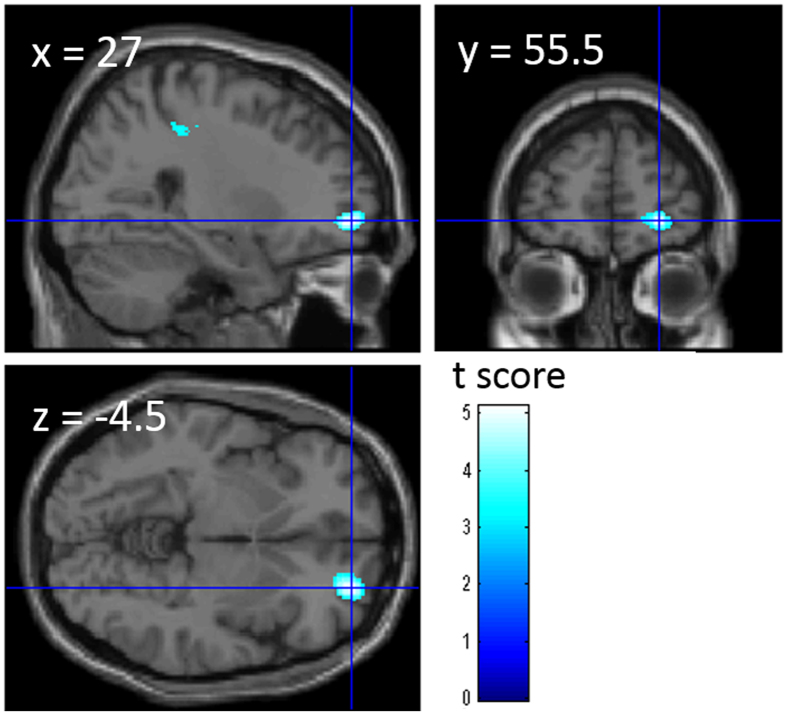
Anatomical correlates of patriotism. The results are overlaid on SPM5’s “single subject” T1-weighted structural image. Blue represents the T score of the negative correlations. Results are shown with *P* < 0.001, uncorrected. A significant negative correlation was observed between patriotism and rGMD in the right RLPFC.

For the rGMD correlates of patriotism, the whole-brain multiple regression analysis showed that the patriotism score was significantly and negatively correlated with rGMD of the anatomical cluster that spread in the rostrolateral prefrontal cortex (RLPFC) (Fig. 2). The results of the statistical analyses are shown in Table 4.

The associations between the mean rGMD of these significant clusters and patriotism or nationalism were relatively weak (r = 0.11–0.18), although the r value became greater when only subjects with extremely high/low (SD > 1.5 or SD < 1.5) patriotism or subjects with extremely high/low nationalism were analyzed (r = 0.23–0.35). Also, the effect size (d) of the differences of mean rGMD between extremely high and low patriotism (or nationalism) were midlevel = (0.50–0.71). For these data, see Supplemental Table 1. But after correcting for age, sex, RAPM score, and TIV, analysis of covariance of the whole brain revealed no significant differences in rGMD between extremely high and low patriotism (or nationalism).

### Post hoc analyses of the associations identified between rGMD of significant clusters and psychological correlates of nationalism and patriotism

Next, to determine the nature of the associations between nationalism, patriotism and the significant clusters identified above, we extracted the mean rGMD from the significant clusters for each individual and investigated the association with other variables on the basis of the hypothesis described above.

Partly consistent with our hypothesis, the mean rGMD of the aforementioned cluster that had a significant correlation with nationalism in the cluster in the amygdala showed a significant negative relationship with suicide ideation and depressive tendency (*P* = 0.014, *t* = −2.452, β = −0.087), but not with the other 10 variables listed in Table 3. The correlation between rGMD in the amygdala and the depression-related trait is consistent with that in our previous study (Takeuchi *et al*. submitted).

In agreement with previous studies, the mean rGMD of the aforementioned significant cluster in OFC had a significant positive relationship with the sense of superiority and the competence factor score (*P* = 0.013, *t* = 2.480, β = −0.093) but not with the 10 other psychological variables in Table 3. The correlation between rGMD in the OFC and the trait related to social comparisons may be consistent with previous studies as described in the Introduction.

In agreement with a previous study^15^ and our hypothesis, the mean rGMD of the aforementioned cluster that had a significant correlation with patriotism in RLPFC showed a significant negative relationship with the average QOL measured by WHOQOL26 (*P* = 0.004, *t* = −2.882, β = −0.103) and Social acceptance vs social intolerance score (*P* = 0.029, *t* = −2.194, β = −0.79), but not with the nine other variables listed in Table 3. The association between regional GM structure and QOL is consistent with the results of our previous study as described in the Introduction.

Partly consistent with our hypothesis, the mean rGMD of the aforementioned cluster that had a significant correlation with nationalism in PCC showed a significant negative relationship with the compassion vs revengefulness factor of cooperativeness (*P* = 0.020, *t* = −2.322, β = 0.085) but not with the other factors related to cooperativeness nor the other psychological variables in Table 3 (10 other variables in total).

## Discussion

To the best of our knowledge, this is the first study to investigate the associations between brain structures and nationalism and patriotism. Partly consistent with our hypothesis, participants with a greater sense of nationalism showed greater rGMD in areas of PCC and OFC and the smaller rGMD of the area in the right amygdala. Participants with greater rGMD in the abovementioned areas of the PCC showed lower compassion (vs. revengefulness). Participants with greater rGMD in the abovementioned areas of OFC showed higher feelings of superiority over others. Participants with greater suicidal tendency showed smaller rGMD in the area of amygdala, but not higher nationalism. Furthermore, partly consistent with aspects of our hypothesis, we demonstrated that participants with higher patriotism showed smaller rGMD in RLPFC. This area’s rGMD and patriotism were also negatively correlated with QOL. Thus, these results indicate that individual nationalism and patriotism are related to the coordination of these (unspecific) multiple cognitive and neural characteristics.

Overall, as discussed below, participants with decreased regional GM structures appeared to show more pro-social cognitive patterns (such as empathy related traits), at least in the case of regional GM within and close to areas around the medial prefrontal and medial parietal areas, which play key roles in social cognition^20^,^44^ (among 567 subjects of this our previous study^20^, 449 subjects overlapped those of the present study). These results are highly consistent with those of previous GM structural studies of young adults^15^,^18^,^45^,^46^,^47^ (among 160 subjects who participated in our previous study ref. 15, 97 overlapped those of the present study; the structural data of all of 185 subjects of our previous study of ref. 18 was used in the present study, too, while subjects in our previous study of ref. 45 did not overlap with those of the present study, and among 303 subjects of our previous study of ref. 46, 185 subjects overlapped those of the present study). As discussed previously^18^, developmental cortical thinning, which is probably caused by synaptic pruning^48^, is observed in these regions after adolescence, and the advanced development of neural systems, which may well be related to mature cognitive abilities in most cases, is characterized by advanced cortical thinning and reduced rGMD. A summary of the associations between the decreased rGMD and increased functioning in these areas can be found in a review by Kanai and Rees^49^.

Our results suggest that participants with higher nationalism show greater rGMD in the area of PCC because participants with lower compassion (vs. revengefulness) had both greater rGMD in this area and higher nationalism. Our psychological results showed that nationalism (but not patriotism) was significantly negatively correlated with cooperativeness, which agrees with previous studies^2^, as well as all of its subfactors. However, the compassion vs revengefulness subfactor had a strong negative correlation with nationalism. Furthermore, participants with higher nationalism and higher compassion (vs revengefulness) (but not other subfactors) showed with smaller rGMD in this area of PCC. This may agree with previous studies, which reported negative correlations with traits related to pro-social characteristics (empathy related traits) and greater regional GM in PCC or the adjacent precuneus areas of healthy young adults^18^,^20^. Functional imaging studies showed that this area is activated by a wide range of social cognition tasks (for review, see ref. 50) and that traits with pro-social characteristics (such as empathy related cognition) have positive correlations with the WM volume in this area of healthy young adults^19^ (among 567 subjects in our previous study^19^, 449 participated in the present study). Our results and previous findings support the interpretation that participants with higher nationalism show greater rGMD in the area of PCC because participants with lower compassion (vs. revengefulness) had both greater rGMD in this area and higher nationalism. These characteristics of cognitive and neural traits may partly underlie a wide range of the psychological characteristics of nationalists including their lack of compassion, e.g., human rights abuses, intolerance to minorities, xenophobia, and ethnic violence, as described in the Introduction.

Our results suggest that participants with higher nationalism showed greater rGMD in the area of OFC because participants with higher feelings of superiority over others showed both higher nationalism and greater rGMD in this area. Our psychological results also found that participants with higher feelings of superiority over others showed higher nationalism, which agrees with the definition of nationalism (a positive evaluation of one’s own group, which is inherently related to derogation of others)^5^. Participants with higher nationalism and those with higher feelings of superiority over others both showed greater rGMD in this area of OFC. This agrees with previous studies, which showed that OFC is particularly important for social comparisons^21^,^51^. Our results and previous findings support the interpretation that participants with higher nationalism showed greater rGMD in the OFC because participants with higher feelings of superiority over others showed both higher nationalism and greater rGMD in this area.

Our results suggest that participants with higher nationalism showed smaller rGMD in the area of the amygdala, while those with suicide ideation did not show higher or lower nationalism, but rather smaller rGMD in the amygdala, suggesting complex associations among these three. Participants with higher nationalism did not show higher or lower suicide ideation and depressive tendency, but participants with smaller rGMD in this area showed higher suicide ideation. Previous neuroimaging studies also showed that these areas are associated with sadness^52^ and a decreased rGMD in these areas in response to depression or stress^53^,^54^. The increased sense of superiority in subjects with higher nationalism described above might erase the association between nationalism, a depressive tendency, and related mechanisms, but subjects with higher nationalism may have neural mechanisms that overlap with those of subjects with suicide ideation. On the other hand, our psychological results also indicated that participants with higher nationalism expressed more traits associated with anger, in agreement with previous psychological studies^4^,^9^. However, in this study, participants with more traits associated with anger did not show greater or smaller rGMD in any cluster. A previous study showed that the disorder characterized by anger showed an increase in regional GM structure in the insula^55^. Perhaps the associations between nationalism and trait anger is mediated by the rGMD of areas other than those identified as significant correlates of nationalism.

Our results suggest that participants with higher patriotism have smaller rGMD in RLPFC, and this may be because participants with higher subjective well-being have higher patriotism and smaller rGMD in RLPFC. Our psychological results showed that subjects with higher QOL exhibited higher patriotism, in agreement with previous psychological studies that showed subjects with higher patriotism had higher attachment to one’s father (which is associated with better moods, higher social skills, less selfishness, and less problematic behaviors)^56^ and feelings of security and self-enhancement^2^,^4^,^9^. Subjects with higher patriotism and those with higher QOL both showed smaller rGMD in this area of RLPFC, which agrees with our previous study^15^ and other neuroimaging studies. The association of this area with positive affects (for summary, see ref. 57) may be mediated by meta-cognitive functions of this area (for summary, see ref. 45). Our results and previous findings support the interpretation that participants with higher patriotism have smaller rGMD in RLPFC, and this may be because participants with higher subjective well-being have higher patriotism and smaller rGMD in RLPFC.

Our psychological results also advanced the understanding of nationalism and patriotism. As described in the Introduction, subjects with higher nationalism have a wide range of political opinions and behaviors involving a competitive or militaristic approach to the world and lack of compassion toward out-groups and minorities, while those with high patriotism generally tended to be opposite. As described in the Introduction, previous studies also showed that nationalists were more aggressive and patriots have a greater attachment to fathers. The present results advance these understandings and showed that, in general, nationalists tend to feel superiority toward others, have generally low cooperativeness-related factors, including empathy, while patriots generally exhibit greater subjective well-being and social acceptance, irrespective of the theme. These findings explain why nationalists and patriots have a wide range of politics-related characteristics and why nations tend to show hostility toward outgroups when they are not fulfilled^58^.

Given the large sample size and marginally significant results in the cluster size test, the present results may seem to suggest a relatively small effect size at least in the given single voxels. However, we emphasize the following points to stress the importance and reliability of our results. First, it is important to recognize that the present results do not indicate that the true effect sizes of the present results are statistically likely to be smaller than those of the other studies in the field (studies of the associations between individual differences of cognition and brain imaging measures). As Vul *et al*.^59^ substantiated, in the whole-brain analyses, due to stringent thresholds and numerous multiple comparisons, observed effect sizes in significant areas are strongly overestimated. In almost all studies in the field, researchers usually show through statistical tests that an effect size is stronger than zero. Furthermore, it has also been shown that statistical power is typically very low even in the field of neuroscience without whole-brain analyses^60^. The consequences of this reality include overestimates of effect size and low reproducibility of results. Even when single studies show a remarkable effect size, under low statistical power in particular, the true effect size can be very small, (for example, ref. 61; thus, the importance of a large sample size has been emphasized^60^). It is important to recognize that under with limited statistical power and sample size, even when the *P* value is the same, more false positives will come up (for the mechanisms of how this happens, please see ref. 60). We did not replicate our findings in this study using multiple samples as is the case with almost all the studies in this field. However, the fact that our study has a larger statistical power alone indicates that our finding is more reliable than other research in the field even when the reported *P* value is the same. Nonetheless, the lack of a strong (or midlevel) correlation between cognition and brain structures of any one area in analyses of a large sample of young adults is a widely seen phenomenon^39^,^62^,^63^,^64^. This phenomenon may suggest that at least in this group, it is difficult to reliably estimate cognitive differences from any one area of one brain image. Thus, to estimate cognitive differences from brain images in this group with the face of this reality, new approaches may be necessary (such as utilization of multiple images and multiple areas).

Nationalism and patriotism are essential individual characteristics that underlie the individuals’ support of important policies, and nationalism is an important factor that has pushed much of the world into chaos and war^2^. In summary, our results indicate that individual nationalism may be mediated by neurocognitive mechanisms in social-related areas and limbic neural mechanisms, whereas patriotism may be mediated by neurocognitive mechanisms in the areas related to well-being. Understanding the neural basis of nationalism and patriotism provides new insights into the nature of nationalism and patriotism, as well as insights into how to understand these characteristics to obtain peaceful societies.

## Additional Information

**How to cite this article**: Takeuchi, H. *et al*. Differences in gray matter structure correlated to nationalism and patriotism. *Sci. Rep.* **6**, 29912; doi: 10.1038/srep29912 (2016).

## Supplementary Material

Supplementary Information

## Figures and Tables

**Table 1:** Demographic variables of the study participants.

Measure	Males		Females	
	Mean	SD	Mean	SD
Age	20.79	1.97	20.57	1.67
RAPM	28.92	3.75	28.27	3.67
Nationalism	19.45	3.94	18.51	3.62
Patriotism	26.97	4.77	26.42	4.40

**Table 2:** Distribution of Nationalism and Patriotism scores of the study participants.

	–9	10–14	15–19	20–24	25–29	30–34	35–
Nationalism (male)	2	34	192	158	44	3	—
Nationalism (female)	5	37	169	117	14	2	—
Patriotism (male)	0	4	20	101	159	131	18
Patriotism (female)	1	3	15	93	144	86	2

**Table 3:** Results of multiple regression analyses between nationalism and patriotism, and other psychological variables (*P* value, *t* value, standardized partial regression coefficient (β)).

	Nationalism				Patriotism			
	*P*, uncorected	*P* (FDR)	*t* value	β^1^	*P*, uncorected	*P* (FDR)	*t* value	β^1^
Average QOL	0.804	0.602	−0.248	−0.010	0.002	0.004	3.121	0.119
Feeling of superiority	3.895*10^−5^	1.145*10^−4^	4.141	0.168	0.955	0.653	−0.057	−0.002
Trait anger	5.762*10^−10^	2.823*10^−9^	6.277	0.237	0.978	0.653	0.028	0.001
Cooperativeness	8.287*10^−12^	6.091*10^−11^	−6.940	−0.258	0.100	0.108	1.647	0.061
Social acceptance vs social intolerance	9.122*10^−7^	3.352*10^−6^	−4.950	−0.189	0.026	0.043	2.238	0.085
Empathy vs social disinterest	0.029	0.042	−2.185	−0.084	0.635	0.546	0.475	0.018
Helpfulness vs unhelpfulness	6.736*10^−5^	1.650*10^−4^	−4.007	−0.155	0.240	0.235	1.177	0.045
Compassion vs revengefulness	1.631*10^−12^	2.398*10^−11^	−7.181	−0.265	0.668	0.546	0.429	0.016
Pure-hearted principles vs self-serving advantage	2.806*10^−4^	5.893*10^−4^	−3.649	−0.139	0.103	0.108	1.630	0.062
Beck Depression Inventory score	0.819	0.602	0.229	0.010	0.369	0.339	−0.900	−0.037
Suicide ideation and depressive tendency	0.093	0.108	1.684	0.066	0.066	0.088	−1.843	−0.071

The multiple regression analyses included age, sex, the RAPM score, and the nationalism and patriotism scores as covariates.

^1^standardized partial regression coefficient.

**Table 4:** Brain regions that had significant correlations with nationalism and patriotism.

Area	x		y	z	T score	Corrected *P*value (cluster)*	Corrected *P*value (SVC, voxel-level FWE)	Cluster size (mm^3^)	Beta**
Positive correlation with nationalism									
OFC	L	−14	63	−29	4.42	0.037	—	2854	0.162
PCC, cerebellum		14	−51	4	3.85	0.050	—	2562	0.158
Negative correlation with nationalism									
Amygdala	R	30	5	−26	3.46	—	0.012	20	−0.131
Negative correlation with patriotism									
RLPFC	R	27	56	−5	5.12	0.033		1249	−0.184

There were no other significant results.

^*^Corrected at the non-stationary cluster size threshold with a voxel-level cluster-determining threshold of *P* < 0.001.

^**^Beta values were for the associations between mean cluster rGMD and nationalism/patriotism after accounting for other covariates in the multiple regression analyses.
