# Supplementary material for: Differences in gray matter structure correlated to nationalism and patriotism
Source: Sci Rep. 2016 Jul 15;6:29912. doi: 10.1038/srep29912 (PMC4945903; doi:10.1038/srep29912)
Supplement: Supplementary Information [file srep29912-s1.doc]

**Supplemental Information**

Differences in gray matter structure correlated to nationalism and patriotism

Hikaru Takeuchi^a^, Yasuyuki Taki^a,b,c^, Atsushi Sekiguchi^b,d^, Rui Nouchi^e^, Yuka Kotozaki^f^, Seishu Nakagawa^d^, Carlos Makoto Miyauchi^d,g^, Kunio Iizuka^d^, Ryoichi Yokoyama^d,h^, Takamitsu Shinada^d^, Yuki Yamamoto^d^, Sugiko Hanawa^d^, Tsuyoshi Araki^f^, Hiroshi Hashizume^a^, Keiko Kunitoki^i^, Yuko Sassa^a^, Ryuta Kawashima^a,d,f^

*^a^Division of Developmental Cognitive Neuroscience, Institute of Development, Aging and Cancer, Tohoku University, Sendai, Japan*

*^b^Division of Medical Neuroimaging Analysis, Department of Community Medical Supports, Tohoku Medical Megabank Organization, Tohoku University, Sendai, Japan*

*^c^Department of Radiology and Nuclear Medicine, Institute of Development, Aging and Cancer, Tohoku University, Sendai, Japan*

*^d^Department of Functional Brain Imaging, Institute of Development, Aging and Cancer, Tohoku University, Sendai, Japan*

*^e^Human and Social Response Research Division, International Research Institute of Disaster Science, Tohoku University, Sendai, Japan*

*^f^Smart Ageing International Research Center, Institute of Development, Aging and Cancer, Tohoku University, Sendai, Japan*

*^g^Graduate Schools for Law and Politics,  The University of Tokyo,  Bunkyo,  Tokyo,  Japan*

*^h^Japan Society for the Promotion of Science, Tokyo, Japan*

*^i^ Faculty of Medicine, Tohoku University, Sendai, Japan*

**Corresponding author:**

Hikaru Takeuchi

Division of Developmental Cognitive Neuroscience, IDAC, Tohoku University

4-1 Seiryo-cho, Aoba-ku, Sendai 980-8575, Japan

Tel/Fax: +81-22-717-7988

E-mail: [takehi@idac.tohoku.ac.jp](mailto:takehi@idac.tohoku.ac.jp)

**Supplemental Methods.**

**The reliability and validity of the National Identity Scale**

The factor structure of this scale has been supported by factor analyses [^1^](#_ENREF_1).The internal consistencies (Cronbach’s alpha) were calculated from the present sample’s data using Predictive Analysis SoftWare release version 22.0.0 (PASW Statistics 22) (SPSS Inc). The internal consistency for nationalism based on the six items was 0.689 and that for patriotism based on the seven items was 0.806, which indicated sufficiently high internal consistency (>0.65 for the standard of ref [^2^](#_ENREF_2)), given the diversity of the items and the number of items. These results indicated the high reliability of this questionnaire. Higher age is associated with higher scores for nationalism and patriotism [^1^](#_ENREF_1). Less knowledge in the international domain is associated with higher nationalism [^1^](#_ENREF_1). Subjects with higher patriotism estimate that there are more patriotic Japanese, whereas subjects with higher nationalism estimate there are more nationalistic Japanese [^1^](#_ENREF_1). These results support the criterion-related validity of the scale.

**The rationales for not including other relevant political-associated measures in this project**

We state that there are no relevant political-associated measures that were not disclosed in this study, other than the National Identity Scale. This is because in Japan, apparently, simple conservatism–liberalism dualism that was previously employed in the UK or US [^3^](#_ENREF_3) does not fit with the political situation. Most political parties cannot be divided into such categories either. Therefore, we did not gather measures of political orientation (conservatism–liberalism). To our knowledge, proper measures for right-wing left-wing distinction were not available in Japan. Asking certain parties for support is a sensitive issue that we would like to avoid. Furthermore, this is a long-lasting cohort study and political parties are changing each year, making it difficult to evaluate the effects of party support in this cohort. Thus, we did not ask which particular parties the subjects supported.

**Other brain images obtained in this project.**

Other than T1-weighted structural images, in this project from the majority of subjects, diffusion-weighted images [^4^](#_ENREF_4), arterial spin labeling images [^5^](#_ENREF_5), resting state functional magnetic resonance imaging (fMRI) scans [^6^](#_ENREF_6), and fMRI scans during the N-back working memory [^7^](#_ENREF_7) were obtained. These images were irrelevant to the purpose of this study and were not used.

**Preprocessing of T1-weighted structural data**

As described in our previous study [^8^](#_ENREF_8), both functional imaging studies and structural studies have advantages and disadvantages, but the findings from the two methods should complement each other. Structural imaging studies are especially useful for investigating the anatomical correlates of personal characteristics involving a wide range of behaviors or opinions that occur outside the laboratory, such as nationalism and patriotism, because unlike fMRI studies, the results of structural imaging studies are not limited to the specific regions engaged in the task or stimuli during scanning. Furthermore, while fMRI studies have certain apparent advantages, one disadvantage is that simplified everyday tasks have to be performed in an MRI scanner because of the constraints of MRI, and this may lead to different activation patterns in the brain [^9^](#_ENREF_9). Furthermore, in MRI correlation studies (including those of fMRI) that investigated the neural basis of individual differences, we are able to use established cognitive measures with proven reliability and validity to tap individual differences in cognition.

As summarized in our previous study [^10^](#_ENREF_10), potential correlates of GM in VBM may include the number and size of neurons and glial cells, the level of synaptic bulk, and the number of neurites, , although this notion remains to be proven by histological studies. GM and structures are known to be associated with various cognitive abilities, and investigation of these associations can identify the brain regions associated with specific cognitive abilities or characteristics (for example, see refs ). Structural imaging thus provides unique and distinctive information about the neural origin of individual cognitive characteristics.

Preprocessing of the structural data was performed using Statistical Parametric Mapping software (SPM8; Wellcome Department of Cognitive Neurology, London, UK) implemented in Matlab (Mathworks Inc., Natick, MA, USA). Using the new segmentation algorithm implemented in SPM8, T1-weighted structural images of each individual were segmented into 6 tissues. In this process, the GM tissue probability map (TPM) was manipulated from maps implemented in the software so that the signal intensities of voxels with (GM tissue probability of the default tissue GM TPM + white matter (WM) tissue probability of the default TPM) < 0.25 became 0.When this manipulated GM TPM is used, the dura matter is less likely to be classified as GM (compared with when the default GM TPM is used), without other substantial segmentation problems. In this new segmentation process, default parameters were used, except that affine regularization was performed with the International Consortium for Brain Mapping template for East Asian brains. We then proceeded to the diffeomorphic anatomical registration through exponentiated lie algebra (DARTEL) registration process implemented in SPM8. In this process, we used DARTEL import images of the 5 TPMs from the abovementioned new segmentation process. First, the template for the DARTEL procedures was created using imaging data from 63 subjects who participated in an experiment in our laboratory [^15^](#_ENREF_15). Next, using this existing template, the DARTEL procedures were performed for all of the subjects in the present study. In these procedures, default parameter settings were used. The resulting images were spatially normalized to the Montreal Neurological Institute (MNI) space to give images with 1.5 × 1.5 × 1.5 mm^3^ voxels. Subsequently, all images were smoothed by convolving them with an isotropic Gaussian kernel of 12 mm full width at half maximum (FWHM) for the reasons described below.

**Areas that were analyzed in the whole brain multiple regression analysis.**

In the whole-brain multiple regression analyses, we included only voxels that showed rGMD values of >0.05 in all subjects. We also removed the lower part of the cerebellum (areas with y < −45 and z < −30) because for some subjects these areas were not included in raw images. The primary purpose for using GM thresholds was to cut the periphery of the GM areas and to effectively limit the areas for analyses. We performed this procedure by limiting the areas for analyses to those likely to be GM. The voxels outside the brain areas are more likely to be affected by signals outside the brain through smoothing. Masking the analysis to brain areas is performed in fMRI analyses of SPM by default. The GM value threshold of 0.05 is a widely used value that has been reported in numerous previous VBM studies .

**Rationale for the method to correct multiple comparisons in the whole brain analysis.**

The statistical significance level was set at *P <* 0.05, corrected at the non-stationary cluster level [^23^](#_ENREF_23) with an underlying voxel level of *P <* 0.001 in the whole brain analysis. In this non-isotropic cluster-size test of random field theory, a relatively higher cluster-determining threshold combined with high smoothing values of more than six voxels leads to appropriate conservativeness in real data. We used the VBM5/SPM5 version of this test and a smoothing value of 12 mm. This is because a previous validation study of this test using a real dataset [^24^](#_ENREF_24) showed that the conditions of this cluster size test are very limited and are dependent on the smoothness of the data, as described above. However, there are substantial differences in the way that SPM8 (or SPM12) and SPM5 estimate actual FWHM in the areas analyzed, and this directly affects the cluster test threshold. Therefore, regardless of whether SPM5 or SPM8 or SPM12 is appropriate, our view is that the conditions for this non-stationary adjusted cluster size test shown by the previous study [^24^](#_ENREF_24) are no longer guaranteed in SPM8 and SPM12 because they are different analyses and produce substantially different results.

**Construction of masks of ROIs.**

All ROIs were constructed using the WFU PickAtlas Tool ([http://www.fmri.wfubmc.edu/cms/software#PickAtlas](http://www.fmri.wfubmc.edu/cms/software#PickAtlas)) and were based on the Brodmann option of the PickAtlas. The masks of the bilateral amygdala were constructed on the basis of this option. The mask of the dorsal ACC area was constructed by using the mask image of regions of Brodmann areas 24.

**Supplemental Discussion**

**The rationales for not including relevant psychological measures in the whole brain multiple regression analysis of patriotism and nationalism.**

We did not include psychological measures related to nationalism and patriotism, in the whole-brain multiple regression analyses, which we used to investigate the associations between nationalism, patriotism, and rGMD. This is because we did not regard these measures as “confounding variables.” This is common in brain imaging correlation analyses of the working memory capacity that do not include psychometric intelligence as a covariate (for example, ref [^27^](#_ENREF_27)), brain imaging analyses of schizophrenia that do not include the working memory capacity as a covariate (for example, ref [^28^](#_ENREF_28)), and whole-brain analyses of depression that do not include neuroticism as a covariate (for example, ref [^29^](#_ENREF_29)). Instead, we regarded these measures, nationalism, and patriotism as the essentially and conceptually common, or partly overlapping, neural and cognitive bases that could not be regressed.

**Discussions regarding nationalism and patriotism should have anatomical correlates.**

Nationalism and patriotism are likely to have an innate biological nature because with political conservatism, which has an essential association with nationalism and patriotism [^30^](#_ENREF_30), genetics accounted for approximately half of the variance [^31^](#_ENREF_31). Political conservatism has also already been shown to be associated with regional gray matter structures [^3^](#_ENREF_3). Further, the basic understanding of these characteristics in the relevant filed are that nationalism and patriotism are the extension of ethnocentrism, in group favoritism, and out-group derogation . Particularly, in group favoritism, and out-group derogation are already known to have neural bases through functional imaging studies and are relatively investigated (for review, see ref [^34^](#_ENREF_34)). In group favoritism, and out-group derogation are both universal and evolved . Experimental studies have demonstrated that ethnocentrism can be easily created through influencing human psychological characteristics [^35^](#_ENREF_35) and that ethnocentrism can evolve [^36^](#_ENREF_36) and can be biologically manipulated through biological treatment (oxytocin inducement) [^37^](#_ENREF_37).

From another perspective, one may say that nationalism and patriotism are likely to be culturally evolved complicated traits that are inherited to a lesser extent. Further, regional GM structure is highly heritable. Thus, the associations between the two are not easily interpreted. However, this idea needs to be viewed with caution. First, consider the notion that political perspective is likely to be inherited to a lesser extent. As described above, a twin study showed that for the overall index of political conservatism which is essentially associated with nationalism and patriotism [^30^](#_ENREF_30), genetics accounted for approximately half of the variance [^31^](#_ENREF_31). The second point is the notion that regional GM structure is highly heritable. A recent study using a large sample dataset of regions of interest showed that the heritability of the GM volume was mostly within the range of 30%–70% [^38^](#_ENREF_38) and actually not so different from most individual differences of behaviors and cognition [^39^](#_ENREF_39). The third point is the notion that one measure is highly heritable so that any investigation into the association of that measure with factors that can be strongly related to cultures may not have much importance. However, the heritability of body mass index was estimated to be approximately 70% [^40^](#_ENREF_40), yet an investigation on cultural factors affecting or changing the body mass index is an important topic in health science.

**Limitation of the present study.**

This study had a few limitations. One was common to our previous studies and other studies that used college cohorts . As discussed previously, we used young healthy subjects with a high educational background. Limited sampling of the full range of intellectual abilities is a common hazard when sampling from college cohorts [^44^](#_ENREF_44). Limited sampling may be an important step to rule out the possibly confounding effects of age or education level that could strongly impact the brain structures and increase the sensitivity of the analyses. However, higher nationalism is associated with a lower education level, older age, and lower income . We were not specifically concerned with the types of anatomical correlates of national identity that covary with age, education level, and income. However, whether the different anatomical correlates exist in samples with different characteristics (children, the old) is an open question, and our findings may not be necessarily generalized to those samples. Thus, our findings should be tested using a wider range of population samples with a normal distribution based on larger and more representative samples. Nationalism and patriotism are associated with a wide range of ideologies. However, some of these associations may depend on the specific nations and cultures [^47^](#_ENREF_47). In contrast to the US [^48^](#_ENREF_48), Japanese nationalism does not seem to be associated with militaristic opinions or (neo)conservativeness [^1^](#_ENREF_1), despite its robust association with aggressiveness in Japan, as demonstrated in the present study (at least at the time of our survey). This may be related to the unique situation in Japan where troops are prohibited by the constitution. Thus, while the essential components of nationalism and patriotism may be same, some of their accompanying cognitive and neural correlates may vary according to the specific nation and culture. These issues may be investigated in future research. Further, because of the fundamental nature of the whole brain analysis of this kind, we could only report strong correlations between structure and nationalism and patriotism that surpassed the stringent threshold or that had a strong a priori hypothesis. However, there may be weakly distributed anatomical correlates all over the brain that we could not report. Finally, our discussion had to rely on reverse inference as were much of the studies in the field.

**Supplemental Table 1.** Effect size of the associations between mean rGMD of the significant clusters and nationalism or patriotism in all samples and in the sample of extreme groups

| Associations | r in all samples | r in the samples of extreme groups* | The standardized mean-difference effect size (d) between extremely high and low groups |
| --- | --- | --- | --- |
| Nationalism and rGMD of cluster of OFC | 0.11 | 0.23 | 0.54 |
| Nationalism and rGMD of cluster of PCC/cerebellum | 0.18 | 0.27 | 0.60 |
| Nationalism and rGMD of the cluster of amygdala | −0.13 | −0.24 | −0.50 |
| Patriotism and rGMD of the cluster of RLPFC | −0.16 | −0.35 | 0.71 |

*In the case of nationalism, 63 subjects with extremely high nationalism scores (SD > 1.5) and 45 with extremely low nationalism scores (SD < 1.5) were included. In the case of patriotism, 47 subjects with extremely high patriotism scores (SD > 1.5) and 43 with extremely low patriotism scores (SD < 1.5) were included.

**References**

1. Karasawa, M. Patriotism, nationalism, and internationalism among Japanese citizens: An etic–emic approach. *Polit. Psychol.* **23**, 645-666 (2002).

2. DeVellis, R.F. Scale development: Theory and applications, Vol. 26. (Sage Publications, Thousand Oaks, CA, 2011).

3. Kanai, R., Feilden, T., Firth, C. & Rees, G. Political orientations are correlated with brain structure in young adults. *Curr. Biol.* **21**, 677-680 (2011).

4. Takeuchi, H. et al. White matter structures associated with empathizing and systemizing in young adults. *Neuroimage* **77**, 222-236 (2013).

5. Takeuchi, H. et al. Cerebral blood flow during rest associates with general intelligence and creativity. *PLoS ONE* **6**, e25532 (2011).

6. Takeuchi, H. et al. Resting state functional connectivity associated with trait emotional intelligence. *Neuroimage* **83**, 318-328 (2013).

7. Takeuchi, H. et al. Associations among imaging measures (2): The association between gray matter concentration and task‐induced activation changes. *Hum. Brain Mapp.* **35**, 185-198 (2014).

8. Takeuchi, H. et al. A voxel-based morphometry study of gray and white matter correlates of a need for uniqueness. *Neuroimage* **63**, 1119-1126 (2012).

9. Okamoto, M. et al. Multimodal assessment of cortical activation during apple peeling by NIRS and fMRI. *Neuroimage* **21**, 1275-1288 (2004).

10. Takeuchi, H. et al. Regional gray and white matter volume associated with Stroop interference: Evidence from voxel-based morphometry. *Neuroimage* **59**, 2899-2907 (2012).

11. May, A. & Gaser, C. Magnetic resonance-based morphometry: a window into structural plasticity of the brain. *Curr. Opin. Neurol.* **19**, 407-411 (2006).

12. Takeuchi, H. et al. Verbal working memory performance correlates with regional white matter structures in the fronto-parietal regions. *Neuropsychologia* **49**, 3466-3473 (2011).

13. Haier, R.J., Jung, R.E., Yeo, R.A., Head, K. & Alkire, M.T. Structural brain variation and general intelligence. *Neuroimage* **23**, 425-433 (2004).

14. Takeuchi, H. et al. Regional gray matter volume of dopaminergic system associate with creativity: Evidence from voxel-based morphometry *Neuroimage* **51**, 578-585 (2010).

15. Takeuchi, H. et al. Failing to deactivate: the association between brain activity during a working memory task and creativity. *Neuroimage* **55**, 681-687 (2011).

16. Nauchi, A. & Sakai, K.L. Greater leftward lateralization of the inferior frontal gyrus in second language learners with higher syntactic abilities. *Hum. Brain Mapp.* **30**, 3625-3635 (2009).

17. White, N.S., Alkire, M.T. & Haier, R.J. A voxel-based morphometric study of nondemented adults with Down Syndrome. *Neuroimage* **20**, 393-403 (2003).

18. Focke, N.K., Thompson, P.J. & Duncan, J.S. Correlation of cognitive functions with voxel-based morphometry in patients with hippocampal sclerosis. *Epilepsy Behav.* **12**, 472-476 (2008).

19. Beal, D.S., Gracco, V.L., Lafaille, S.J. & De Nil, L.F. Voxel-based morphometry of auditory and speech-related cortex in stutterers. *Neuroreport* **18**, 1257-1260 (2007).

20. Mueller, S.G. et al. Voxel-based Optimized Morphometry (VBM) of Gray and White Matter in Temporal Lobe Epilepsy (TLE) with and without Mesial Temporal Sclerosis. *Epilepsia* **47**, 900-907 (2006).

21. Schaufelberger, M.S. et al. Grey matter abnormalities in Brazilians with first-episode psychosis. *The British Journal of Psychiatry* **191**, s117 (2007).

22. Takeuchi, H. et al. Regional gray matter density associated with emotional intelligence: Evidence from voxel-based morphometry. *Hum. Brain Mapp.* **32**, 1497-1510 (2011).

23. Hayasaka, S., Phan, K.L., Liberzon, I., Worsley, K.J. & Nichols, T.E. Nonstationary cluster-size inference with random field and permutation methods. *Neuroimage* **22**, 676-687 (2004).

24. Silver, M., Montana, G. & Nichols, T.E. False positives in neuroimaging genetics using voxel-based morphometry data. *Neuroimage* **54**, 992-1000 (2012).

25. Maldjian, J.A., Laurienti, P.J. & Burdette, J.H. Precentral gyrus discrepancy in electronic versions of the Talairach atlas. *Neuroimage* **21**, 450-455 (2004).

26. Maldjian, J.A., Laurienti, P.J., Kraft, R.A. & Burdette, J.H. An automated method for neuroanatomic and cytoarchitectonic atlas-based interrogation of fMRI data sets. *Neuroimage* **19**, 1233-1239 (2003).

27. Hampson, M., Driesen, N.R., Skudlarski, P., Gore, J.C. & Constable, R.T. Brain connectivity related to working memory performance. *J. Neurosci.* **26**, 13338 (2006).

28. Honea, R., Crow, T.J., Passingham, D. & Mackay, C.E. Regional deficits in brain volume in schizophrenia: a meta-analysis of voxel-based morphometry studies. *A. J. Psychiatry* **162**, 2233-2245 (2005).

29. Peng, J. et al. Cerebral and cerebellar gray matter reduction in first-episode patients with major depressive disorder: a voxel-based morphometry study. *Eur. J. Radiol.* **80**, 395-399 (2011).

30. Schatz, R.T., Staub, E. & Lavine, H. On the varieties of national attachment: Blind versus constructive patriotism. *Polit. Psychol.* **20**, 151-174 (1999).

31. Alford, J.R., Funk, C.L. & Hibbing, J.R. Are political orientations genetically transmitted? *American political science review* **99**, 153-167 (2005).

32. Rushton, J.P. Ethnic nationalism, evolutionary psychology and Genetic Similarity Theory*. *Nations Natl.* **11**, 489-507 (2005).

33. Lockyer, A. & Hatemi, P.K. Resolving the difference between evolutionary antecedents of political attitudes and sources of human variation. *Canadian Journal of Political Science* **47**, 549-568 (2014).

34. Molenberghs, P. The neuroscience of in-group bias. *Neurosci. Biobehav. Rev.* **37**, 1530-1536 (2013).

35. Efferson, C., Lalive, R. & Fehr, E. The coevolution of cultural groups and ingroup favoritism. *Science* **321**, 1844-1849 (2008).

36. Fu, F. et al. Evolution of in-group favoritism. *Scientific reports* **2**, Article number: 460 (2012).

37. De Dreu, C.K., Greer, L.L., Van Kleef, G.A., Shalvi, S. & Handgraaf, M.J. Oxytocin promotes human ethnocentrism. *Proc. Natl. Acad. Sci. U. S. A.* **108**, 1262-1266 (2011).

38. Winkler, A.M. et al. Cortical thickness or grey matter volume? The importance of selecting the phenotype for imaging genetics studies. *Neuroimage* **53**, 1135-1146 (2010).

39. Ando, J. Psychology of genetic and environment (iden-to-kankyo-no-shinrigaku) - introduction to human behavior genetics (ningen-koudo-idengaku-nyumon) (world of psychology (shinrigaku-no-sekai ―expert course (senmon-hen)) (Baifukan, Tokyo, Japan, 2014).

40. Elks, C.E. et al. Variability in the heritability of body mass index: a systematic review and meta-regression. *Frontiers in endocrinology* **3**, article 29 (2012).

41. Takeuchi, H. et al. Failing to deactivate: the association between brain activity during a working memory task and creativity. *Neuroimage* **55**, 681-687 (2011).

42. Takeuchi, H. et al. White matter structures associated with creativity: Evidence from diffusion tensor imaging. *Neuroimage* **51**, 11-18 (2010).

43. Song, M. et al. Brain spontaneous functional connectivity and intelligence. *Neuroimage* **41**, 1168-1176 (2008).

44. Jung, R.E. et al. Neuroanatomy of creativity. *Hum. Brain Mapp.* **31**, 398-409 (2010).

45. Coenders, M., Gijsberts, M. & Scheepers, P. [Chauvinism and patriotism in 22 countries] Nationalism and Exclusion of Migrants: Cross-National Comparisons. [M. Gijsbersts, L. Hagendoorn & P. Scheepers (ed.)] [29-69] (Aldershot, Ashgate, 2004).

46. Hjerm, M. & Schnabel, A. Mobilizing nationalist sentiments: Which factors affect nationalist sentiments in Europe? *Soc. Sci. Res.* **39**, 527-539 (2010).

47. Balabanis, G., Diamantopoulos, A., Mueller, R.D. & Melewar, T. The impact of nationalism, patriotism and internationalism on consumer ethnocentric tendencies. *Journal of International Business Studies* **32**, 157-175 (2001).

48. Kosterman, R. & Feshbach, S. Toward a measure of patriotic and nationalistic attitudes. *Polit. Psychol.* **10**, 257-274 (1989).
